# Symptom and Handicap Severity in Subacute Dizzy Patients: The Role of Gaze Position Error in Video Head Impulses

**DOI:** 10.1097/MAO.0000000000004579

**Published:** 2025-09-12

**Authors:** Athanasia Korda, Thomas Wyss, Ewa Zamaro, Efterpi Michailidou, Tom Gawliczek, Franca Wagner, Marco D. Caversaccio, Georgios Mantokoudis

**Affiliations:** ∗ Department of Otorhinolaryngology, Head and Neck Surgery, Inselspital, University Hospital Bern and University of Bern; † University Institute of Diagnostic and Interventional Neuroradiology, Inselspital, University Hospital Bern and University of Bern, Bern, Switzerland

**Keywords:** Acute unilateral vestibulopathy, Dizziness, Dizziness Handicap Inventory, Gaze position error, Quality of life

## Abstract

**Objective:**

This study aims to investigate the significance of gaze position error (GPE) after rapid head movements in the recovery of symptoms among patients with acute unilateral vestibulopathy (AUVP).

**Methods:**

We studied 26 patients with AUVP and a control group of 48 healthy subjects. All patients received a video head impulse test (vHIT) during the acute stage and a follow-up vHIT 30 days after symptoms onset. Additionally, they completed a Dizziness Handicap Inventory (DHI) questionnaire.

**Results:**

Based on our normative data, we found a cutoff amplitude of 4 degrees for abnormal GPE. Mean DHI score was 26.7 (±28.9 SD). Seventeen patients had a mild, four had a moderate, and five a severe dizziness handicap. DHI was highly correlated with GPE (adjusted *R*^2^ = 0.446) and with the vestibulo-ocular reflex (VOR) gain (adjusted *R*^2^ = 0.272). Although age and gender were not significantly associated with DHI, there was a trend toward lower DHI scores in males. In the subscale analysis, we found a marginal correlation between females and emotional DHI subscores and between age and functional DHI subscores (*p* < 0.05).

**Conclusions:**

Our study revealed a correlation between gaze position error (GPE) during rapid head movements and subacute symptoms after AUVP, explaining nearly 50% of the variance in Dizziness Handicap Inventory (DHI) scores. Additionally, emotional symptoms exhibited a gender bias, predominantly affecting females, whereas functional symptoms showed a slight dependence on age.

## INTRODUCTION

Acute unilateral vestibulopathy (AUVP) manifests as an acute vestibular syndrome (AVS) due to a sudden unilateral loss of peripheral vestibular function without any concurrent central or audiological symptoms or signs (1). After the acute stage of AUVP, patients invariably improve. Although many patients achieve symptom relief through central vestibular adaptation and compensation, research indicates that approximately 50% of patients continue to suffer from chronic dizziness, disequilibrium, and spatial disorientation (2,3) (4).

It has been suggested that a persistent peripheral vestibular loss in the chronic phase (beyond 3 mo) might account for the enduring symptoms of AUVP (5). Standard measures for assessing peripheral vestibular loss include semicircular canal function evaluations via bithermal caloric stimulation and video head impulse tests (vHIT). However, previous research has demonstrated that the low-frequency vestibulo-ocular reflex (VOR) response elicited by caloric tests does not predict the severity of chronic dizziness or vertigo symptoms (6). Furthermore, recent studies (7) (8) (9) have shown no significant correlation between high-velocity VOR gain in vHIT and the severity of symptoms post-AUVP, or with other vestibular assessments such as vestibular evoked myogenic potentials (VEMPs) and posturography (10). However, Lee et al. suggested that specific vHIT parameters, such as covert saccades, may be predictive of residual symptoms after a peripheral vestibular deficit (11).

A high ratio of covert saccades in vHIT is associated with improved performance in dynamic visual tasks, gait, and balance assessments (12). Additionally, well-organized corrective saccades, occurring in a clustered pattern with consistent latency after each impulse, are correlated with lower Dizziness Handicap Inventory (DHI) scores (13). Covert saccades, which occur during head movements, ensure that the visual target is projected onto the macula, facilitating clear vision. These corrections are believed to minimize the gaze position error (GPE) (14), defined as the residual difference between the earth-fixed visual target and the actual eye position at the end of the head impulse. The benefit of measuring GPE rather than assessing VOR gain or saccades separately is that GPE captures the combined error from both slow-phase eye velocity (i.e., VOR gain) and any inaccurate, imprecise covert saccades. As a result, GPE provides a more comprehensive and functional measure of gaze stability after head movement compared with analyzing gain or saccades individually (15). Unlike VOR measures, GPE is not currently calculated or analyzed by available vHIT devices.

Although numerous studies have investigated the role of corrective saccades in patients experiencing chronic symptoms after AUVP, there is a paucity of research on GPE measured by a vHIT in the acute and subacute stage. Therefore, in this study, we sought to test whether residual symptoms post-AUVP correlate with GPE from vHIT recordings.

## MATERIALS AND METHODS

### Healthy Subjects

We recruited healthy participants with no history of vestibular disorders. Each participant underwent a vHIT to determine normative GPE values. The mean GPE values were calculated, and the upper cutoff threshold was established at two standard deviations above the mean.

### Patients

We recruited AVS patients as part of a prospective cross-sectional study of patients seen for vertigo in the emergency department (ED) (DETECT—Dizziness Evaluation Tool for Emergent Clinical Triage) between July 2015 and April 2020. We included only patients with a diagnosis of AUVP. The diagnosis was based on the clinical history, vestibular function tests (unilateral hypofunction in caloric test or pathologic vHIT), and audiometry and negative MRI DWI 72 hours after the onset of symptoms to exclude a central disorder. Inclusion criteria were continuous dizziness, with nausea or vomiting, head-motion intolerance, new gait or balance disturbance, and spontaneous nystagmus. We excluded patients younger than 18 years, if symptoms lasted <24 hours, or if the index ED visit was >72 hours after the onset of symptoms. All patients were scheduled for follow-up 1 month after symptoms onset (16). At the follow-up, the patients received vHIT and completed a DHI questionnaire in German language (17).

### Questionnaires

Symptom severity was measured with DHI, a German-validated version of a 25-item questionnaire for assessing physical and emotional symptoms and functional impairment due to dizziness (9 functional questions, 9 emotional questions, and 7 physical questions) (18) (19). Questions are answered as “0” (never), “2”(sometimes), and “4” (always), giving a total score of 0 to 100, with subscale scores of 36, 36, and 28. Scores between 0 and 29 represent a mild dizziness handicap, 30 to 60 a moderate handicap, and >60 a severe handicap (18) (19).

### Video Head Impulse Test

**FIG. 1:**
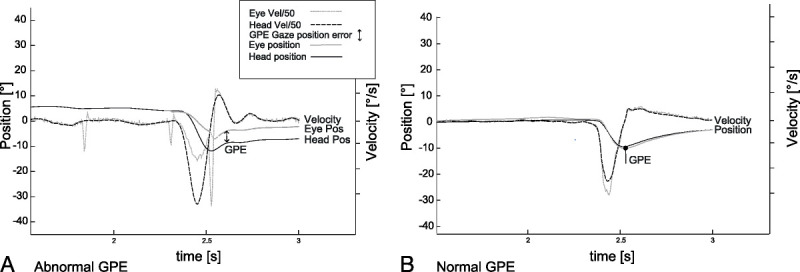
Example of a video head impulse test, which shows an abnormal gaze position error (GPE; *A*) and a normal GPE (*B*). The black line shows the head position, and the gray dotted line the eye position. The black dotted line shows the head velocity, and the light gray dotted line shows the eye velocity. Gaze position error is shown by the arrow.

vHIT was performed by fast passive horizontal head movements (high frequency, 10- to 20-degree head excursion in 100–300 ms corresponding to a 1,000- to 6,000-deg/s^2^ acceleration) in room light during visual target fixation at >1 m distance. We recorded head and eye movement velocity with a head-mounted infrared high-speed camera (EyeSeeCam, Munich) connected to a laptop by USB (20). VOR gain was calculated as the ratio of the median of eye and head velocity of the accepted trials in a window between 55 and 65 milliseconds after the onset of the head impulse (instantaneous VOR gain at 60 ms). We determined the head position by the integration of head velocity signals (Fig. 1, A and B) and calculated the GPE by subtracting eye position from head position at the time point where head velocity reached 0 deg/s at the end of the head impulse (first zero crossing of the head velocity trace) (Fig. 1). All data were analyzed using custom-written code in MatLab Script (2022 MathWorks).

### Statistics

All statistics were reported using the SPSS statistical software (IBM SPSS Statistics for Windows, Version 25.0.; IBM Corp., Armonk, NY). We tested our normative data for normality with the Shapiro-Wilk test. We performed univariate and multivariate regression models to analyze demographic and dizziness-related characteristics (age, gender, lesion side, VOR gain, GPE) that accounted for the degree of variance in the DHI score. Linear correlation coefficients were provided for numerical data as adjusted *R*^2^ values. Mann-Whitney *U* tests were performed to test for differences in categorical data (gender, lesion side). In all the statistical analyses, a value of *p* < 0.005 was considered statistically significant.

### Ethics

All enrolled patients gave written consent. The local ethics committee (IRB) approved this study.

## RESULTS

### Normative Data

We analyzed data from 47 healthy subjects including 32 women and 15 men (age range, 21–81 yr; mean, 43 ± 17 yr). GPE was normally distributed as showed by the Shapiro-Wilk test of normality (*p* > 0.05). Based on our normative data, we considered a cutoff >3.98 degrees for right and 3.97 degrees for left deficits (two standard deviations from the mean) as an abnormal GPE.

### Patients

We analyzed data from 26 patients (10 women and 16 men aged between 30 and 77 yr; mean, 54 ± 14 yr) with a diagnosis of AUVP who received a follow-up vHIT 30 days after symptoms onset. Eleven had a left-side and 15 a right-side vestibular deficit. The mean DHI score was 26.7, with a standard deviation of 28.9. Seventeen patients had a mild dizziness handicap, four had a moderate, and five had a severe handicap. Patients' characteristics are summarized in Table 1. Figure 1A depicts an example of vHIT recordings in a patient with AUVP.

**TABLE 1:** Correlation of DHI scores to patient characteristics and vHIT parameters

Characteristic	Mean (SD) or Percentage	Mean DHI Scores	*p* Value	Adjusted *R*^2^
Age, y	54 (14)	26.7	0.057*^a^*	
Gender				
Female	38%	37	0.060*^b^*	
Male	62%	20		
Deficit side				
Right	43%	23	0.799*^b^*	
Left	57%	29		
VOR gain at 30 d	0.73		0.002	0.304*^c^*
GPE (deg) at 30 d	3.12		<0.001	0.446*^c^*

*^a^* Pearson correlation (one-sided).

*^b^* Mann-Whitney.

*^c^* ANOVA.

### VOR Gain and GPE Improvement Over Time

Over a period of 30 days, mean VOR gain improved from 0.43 to 0.73 and mean GPE decreased from 5.44 to 3.12 degrees. 46% (12 of 26) of the AUVP patients normalized their VOR gain (>0.8), whereas 69% (18 of 26) normalized their GPE (<4 degrees) 30 days after symptoms onset.

### Analysis of Parameters Predicting DHI Score

**FIG. 2:**
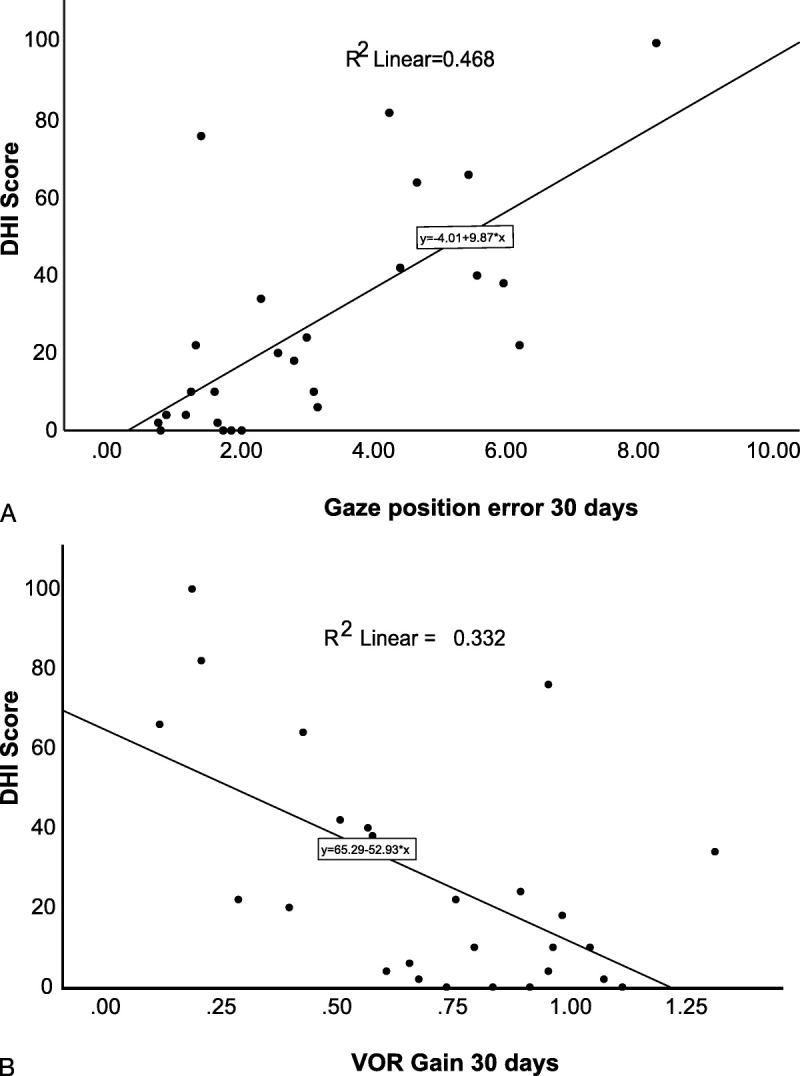
*A*, Correlation of DHI score to GPE in 30 days. There is a positive correlation. High GPE is associated with higher DHI scores (*p* < 0.001). *B*, Correlation of DHI score to VOR gain in 30 days. There is a negative correlation. High gains are associated with lower DHI scores (*p* = 0.002).

**FIG. 3:**
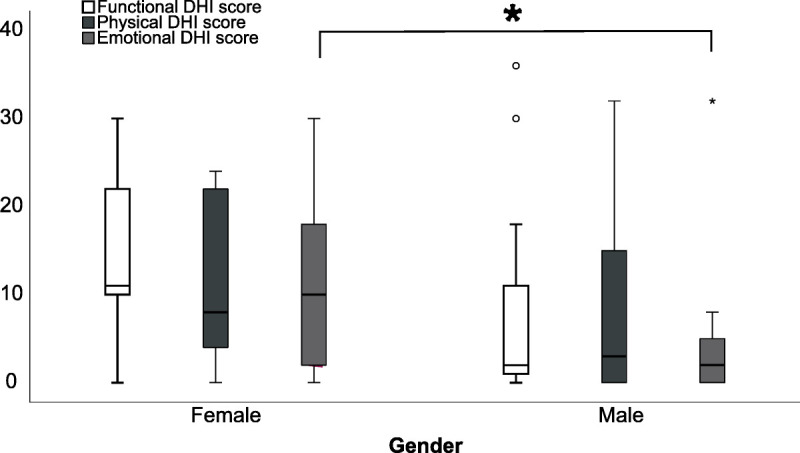
Box-plot whiskers of the DHI subscales for males and females. Emotional DHI score is statistically significant different between males and females (*p* = 0.023).

Table 1 shows the factors found to correlate most highly with DHI score variability. We found that GPE after 30 days was the most highly correlated factor on univariate analysis, with an adjusted *R*^2^ value of 0.446 (*p* < 0.001; Fig. 2A). VOR gain at 30 days showed a significant correlation with DHI scores (adjusted *R*^2^ = 0.304, *p* = 0.002), as illustrated in Figure 2B. VOR gain in the acute phase, however, did not significantly correlate with DHI (adjusted *R*^2^ = 0.148, *p* = 0.030). Although age and gender were not significantly associated with DHI scores, there was a trend toward lower DHI scores in males (*p* = 0.060, Table 1). In the subscale analysis of the emotional, physical, and functional parts of DHI, we found a marginal correlation between females and emotional DHI score (*p* < 0.05; Table S2, http://links.lww.com/MAO/C129; Fig. 3) and between age and functional DHI score (*p* < 0.05, Table 2). Patients with left-sided deficits exhibited marginally higher DHI scores on average (23 versus 29) compared with others; however, this difference was not statistically significant.

**TABLE 2:** Cross-tabulation of different GPE and VOR gain combinations in patients with mild dizziness (DHI <30) and severe dizziness (DHI >30)

			Gaze Position Error (GPE)	
	Handicap	Test	Normal	Abnormal
**VOR gain**	Mild n = 17	Normal	10 (38%) recovered	0 (0%)
		Abnormal	6 (23%) compensated	1 (4%) uncompensated
	Severe n = 9	Normal	2 (8%) recovered with symptoms	0 (0%)
		Abnormal	0 (0%)	7 (27%) uncompensated
	n = 26	Total	18	8

Given the significant correlations of these factors with (DHI) scores observed in the univariate analysis, we constructed a multivariate model that included all these factors. This model accounted for a substantial portion of the variance in DHI scores. Our analysis revealed that gender, VOR gain, and GPE collectively explained 50.7% of the variance in DHI scores (adjusted *R*^2^ = 0.507, *p* < 0.001).

## DISCUSSION

Our research demonstrates that gaze position error after rapid head movements is associated with subacute symptoms in patients with acute unilateral vestibular deficits, accounting for nearly 50% of the variance in Dizziness Handicap Inventory scores. Additionally, our findings indicate a gender-based variation in emotional symptoms, with females experiencing a greater impact, and a slight age dependence in functional symptoms.

### Covert Saccades and GPE

Patients with unilateral vestibular deficits were observed to initiate saccades during head movements toward the affected side to reorient their gaze on target. This mechanism allows them to reduce the number of additional overt saccades when the head stands still. These “covert” saccades, which occur during head rotation, effectively compensate for the inadequacy of their VOR. Such covert saccades might obscure a complete unilateral or bilateral loss of canal function. It has been demonstrated that compensatory saccades “covert” reduce GPE during active and passive head impulses; whereas, corrective saccades “overt” do not (21). Moreover, DHI score was positively correlated with velocity of covert saccades 6 months after the acute stage (22). In a study of Riska et al. (12), which examined the correlation of covert saccades to DHI scores, no significant relationship was found. However, they did observe differences in gait and fall risk based on the type of saccade latency. The discrepancy between our study and theirs may be attributed to their inclusion of patients with peripheral deficits and varying symptom durations, whereas we assessed all patients at a consistent time point post-symptom onset.

### Discrepancy Between VOR Gain and GPE

Our findings highlight cases where GPE remains normal despite abnormal VOR gain. VOR gain, measured as velocity gain at 60 milliseconds post-head movement onset, reflects the eye's ability to match head velocity during motion. In contrast, GPE quantifies the positional deviation between the visual target and eye position at the end of head movement.

A key factor in this discrepancy is the role of corrective saccades, particularly compensatory saccades occurring after 60 milliseconds but before the head stops moving. These saccades can effectively reduce GPE, even in cases of reduced VOR gain, by realigning gaze with the target before the head movement ends (15). This suggests that some patients with vestibular dysfunction develop compensatory mechanisms that mitigate gaze instability, leading to a normal GPE despite impaired VOR function. Notably, all patients in our study with normal VOR gain also exhibited normal GPE, reinforcing the expectation that an intact VOR system ensures accurate gaze stability without the need for additional corrective mechanisms.

However, this relationship does not always hold in neurological conditions affecting vestibular and ocular motor integration. Patients with concussion, cerebellar disorders, or neurodegenerative diseases may exhibit normal or near-normal VOR gain yet present with large GPE, likely due to faulty central processing of vestibular and visual information. This suggests that VOR gain alone may not fully capture gaze instability in patients with central vestibular dysfunction, making GPE a valuable complementary measure in assessing gaze control.

These findings emphasize the importance of evaluating both VOR gain and GPE to distinguish between peripheral vestibular dysfunction, adaptive compensatory mechanisms, and central integration deficits. A comprehensive approach incorporating both measures can enhance clinical assessment and guide targeted interventions for patients with dizziness and gaze instability.

### Progression Patterns of Vestibular Neuritis

After acute vestibular neuritis, we identified four distinct patterns of vestibulo-ocular reflex (VOR) gain and gaze position error (GPE) outcomes, namely, 1) unrecovered compensated state, 2) unrecovered uncompensated state, 3) recovered without symptoms, and lastly 4) recovered with symptoms (Table 2).

Approximately 38% of patients exhibited complete recovery, characterized by normal VOR gain and GPE, presenting no residual symptoms. Conversely, 23% of patients demonstrated compensated deficits, maintaining normal GPE despite abnormal VOR gain, often eliciting covert saccades during rapid head movements, indicating that VOR gain alone may not reliably assess compensation.

Two patient groups showed uncompensated deficits: 4% experienced mild dizziness, and 27% suffered severe dizziness, both groups exhibiting abnormal VOR gain and GPE. Additionally, 8% of patients presented with severe symptoms despite normal VOR gain and GPE.

### Risk Factors for Symptom Persistence and Severity

We found that females are more likely to develop emotional symptoms after AUVP. There are studies that support this finding as they have shown that the female gender represents a risk factor in the development of chronic vertigo mostly because of their high somatic anxiety scores (19) (23). Age is also a potential important factor that accounts for the chronic subjective impairment after vestibular deficits. In our cohort, we showed a significant correlation only to the functional symptoms. There are studies that support perceived symptoms (24) or balance control (25) after AUVP as a function of age. Patients with left-sided vestibular deficits tend to have higher DHI scores. A mouse model study demonstrated that right- and left-sided unilateral vestibular deficits differently affect spatial cognition and locomotion, leading to distinct compensatory patterns (26). To our knowledge, it has been not yet studied in humans if the side of the deficit is a parameter that influences compensation after AUVP. More studies needed to test this hypothesis.

### Strengths and Limitations

Although our study provides novel insights into the relationship between GPE and subacute symptoms after AUVP, several limitations should be acknowledged. First, our sample size was relatively small (n = 26), which may limit the generalizability of our findings. A larger cohort could provide more robust statistical power and allow for subgroup analyses based on factors such as lesion side and compensation mechanisms. Second, we did not assess vestibular function beyond the horizontal semicircular canal. The anterior and posterior canals, as well as otolith function (saccule and utricle), were not evaluated, which may have contributed to unexplained variance in Dizziness Handicap Inventory (DHI) scores. Future studies should incorporate comprehensive vestibular assessments to better capture the full spectrum of vestibular impairment. However, a study by Möhwald et al. (27) demonstrated that vestibular-ocular motor disturbances in the yaw plane have a greater impact on DHI scores than vestibular-spinal or vestibular-perceptive asymmetries in the roll and pitch planes. This suggests that horizontal visual stability is the most critical parameter influencing DHI scores. Third, although we demonstrated a correlation between GPE and dizziness-related handicap, we did not analyze additional compensatory mechanisms such as the latency, amplitude, or frequency of covert saccades, which could further refine our understanding of gaze stabilization strategies. Including these parameters in future studies may improve predictive models for symptom persistence. Fourth, the use of custom-written MATLAB code for GPE analysis introduces a methodological limitation, as current commercially available vHIT systems do not offer automated GPE quantification. Standardizing GPE measurements across different devices and software would enhance reproducibility and clinical applicability. Fifth, we did not systematically document whether patients wore corrective vision glasses or contact lenses before testing, which may have influenced our results. This factor should be controlled for in future studies to ensure methodological consistency. Finally, our study focused on the subacute stage of AUVP, limiting its applicability to patients with chronic vestibular symptoms. Further research should investigate whether GPE remains a relevant marker of persistent dizziness beyond the 1-month follow-up period. Despite these limitations, our findings highlight the potential clinical utility of GPE for symptom severity in dizzy patients and underscore the need for further research to optimize vestibular assessment and rehabilitation strategies.

### Clinical Implications

Our study underscores the importance of measuring GPE to account for subacute symptoms after AUVP. We identified four distinct patterns of disease progression that have significant implications for patient management.

Patients classified as unrecovered and uncompensated exhibit both abnormal VOR gain and abnormal GPE (GPE > 4). These patients require intensive vestibular physiotherapy, with early intervention (within 2 wk) being crucial to prevent the progression to chronic disease (28). Recovered but symptomatic patients might be prone to develop a chronic vestibular syndrome with increasing tendency of avoidance and permanent gait instability or persistent dizziness. Such patients may be classified as having PPPD if symptoms persist beyond 3 months.

Our findings suggest that calculating both VOR gain and GPE provides complementary insights into vestibular compensation, partially explaining the associated residual handicap. Relying solely on VOR gain may be misleading, as patients with identical VOR gain values can exhibit variable GPE and, consequently, different symptoms and progression patterns.

Additionally, female gender and advanced age emerged as important risk factors for symptom persistence and elevated dizziness handicap scores after unilateral vestibular loss. We recommend initiating early gaze stabilization exercises, particularly in older female patients, as vestibular rehabilitation is regarded as the gold standard treatment (29). Future studies but also future vHIT devices should incorporate automated analysis of GPE to enhance diagnostic accuracy and treatment efficacy.

## CONCLUSIONS

Our study identified a correlation between gaze position error (GPE) during rapid head movements and subacute symptoms after AUVP, accounting for nearly 50% of the variance in Dizziness Handicap Inventory (DHI) scores. We delineated four distinct disease progression patterns in the subacute stage, enabling more targeted patient management. Furthermore, emotional symptoms displayed a gender bias, predominantly affecting females, whereas functional symptoms exhibited a slight age dependence.
